# Combination of adipose-derived stem cell conditioned media and minoxidil for hair regrowth in male androgenetic alopecia: a randomized, double-blind clinical trial

**DOI:** 10.1186/s13287-023-03440-2

**Published:** 2023-08-21

**Authors:** Lili Legiawati, Lis Surachmiati Suseno, Irma Bernadette S. Sitohang, Shannaz Nadia Yusharyahya, Jeanne Adiwinata Pawitan, Isabella Kurnia Liem, Trie Kurniawati, Athaya Ardelia, Kanya Paramastri

**Affiliations:** 1 Department of Dermatology and Venereology, Faculty of Medicine, Dr. Cipto Mangunkusumo Hospital, Universitas Indonesia, Diponegoro No. 71, Central Jakarta, DKI Jakarta 10430 Indonesia; 2 Stem Cell Medical Technology, Integrated Service Unit, Faculty of Medicine, Dr. Cipto Mangunkusumo Hospital, Universitas Indonesia, Jakarta, Indonesia; 3 Department of Histology, Faculty of Medicine, Universitas Indonesia, Jakarta, Indonesia; 4 Faculty of Medicine, Stem Cell and Tissue Engineering Research Center, Indonesia Medical Education and Research Institute (IMERI), Universitas Indonesia, Jakarta, Indonesia

**Keywords:** Adipose tissue-derived stem cells conditioned media, Alopecia, Androgenetic alopecia, Hair regrowth, Minoxidil, Stem cells

## Abstract

**Introduction:**

Treatments for AGA have yet to produce satisfactory outcomes and may cause intolerable side effects. Recent studies have reported that adipose tissue-derived stem cell conditioned media (ADSC-CM) could induce hair growth and regeneration.

**Objective:**

To investigate the efficacy of ADSC-CM combined with minoxidil for hair regeneration therapy in male AGA.

**Methods:**

This study lasted for 6 weeks. Subjects were divided into two groups: concentrated and non-concentrated ADSC-CM. Scalp was divided vertically in half before intradermal injection was administered from the frontal region of the scalp toward the vertex with a 30G needle, spaced about 1 cm apart. Treatment side received 2 ml of ADSC-CM; the other side was given 2 ml of NaCl 0.9% as placebo. Patients applied 5% minoxidil twice daily post-injection. Improvements were assessed using photographs and trichoscan every 2 weeks.

**Results:**

Hair count, hair density, and mean thickness increased significantly on both sides after 6 weeks, while vellus rate decreased proportionally with the increase of terminal rate. No statistically significant differences between treatment groups were found. Minimum side effects were reported, and subjects were satisfied with the results.

**Conclusion:**

Combination of ADSC-CM and minoxidil could be a potential agent for hair regrowth. Follow-up research with extensive populations, longer duration, and different study design may be required to confirm the exact mechanisms of ADSC-CM on hair growth.

*Trial registration*: Clinicaltrials.gov, NCT05296863. Registered 25 March 2022—Retrospectively registered, https://clinicaltrials.gov/ct2/show/NCT05296863.

**Supplementary Information:**

The online version contains supplementary material available at 10.1186/s13287-023-03440-2.

## Introduction

Alopecia is classified into two categories, which are cicatricial and noncicatricial alopecia [1, 2]. Noncicatrical alopecia is more common, with androgenetic alopecia (AGA) as the most encountered subtype in clinical settings. The development of AGA is attributed to excessive secretion of dihydrotestosterone (DHT) and genetic factors, causing a patterned hair loss as well as miniaturization of hair follicles [3]. In males, this disorder manifests as regression in the frontotemporal hairline and balding of the vertex. On the other hand, female pattern hair loss displays as dispersed thinning of the hair with a preserved hairline [4].

Although it is also encountered in females, AGA is more prevalent in males of various ages. AGA manifests after puberty and progressively worsens with age [1, 2]. The age of onset occurs between 20–25 years in males and 40–50 years in females [2]. While AGA is not life-threatening, most patients experience a significant decrease in self-esteem, particularly physical appearance. According to a meta-analysis conducted by Huang et al., AGA is heavily related to a decline in health-related quality of life (HRQOL) and associated with the occurrence of psychological disorders [5].

There are currently few AGA treatments available, yet little have met expectations [4, 6]. The first-line treatment for AGA is oral finasteride and topical minoxidil. Despite the fact that these substances can cause a variety of serious local and systemic side effects, both of these therapies have been approved by the Food and Drug Administration (FDA) and proven to be effective [4]. Early treatment initiation, as well as uninterrupted treatment continuation, may delay disease progression and maintain favorable results. Hence, patient compliance is extremely important in determining the outcomes. As the pathophysiology of AGA is multifactorial, therapy using multiple modalities targeted at different mechanisms is recommended.

The development of biotechnologies to enhance hair regrowth in AGA is clinically necessary. This implies that the aim of hair tissue engineering (HTE) must be the invention of new autologous technologies involving hair regrowth. In order to promote hair development, autologous stem cells have attracted a lot of attention. As a potential treatment for AGA, platelet-rich plasma (PRP) and micrografts containing human follicular mesenchymal stem cells (HF-MSCs) were tested [7]. In addition, the ability of autologous platelet-derived growth factors to enhance cell proliferation, differentiation, and neo-angiogenesis, supporting the wound healing process, can be a major reason for their application in hair tissue regeneration (HTR). In actual fact, PRP contains at least six substantial growth factors, including the basic fibroblast growth factor (bFGF), platelet-derived growth factor (PDGF), vascular endothelial growth factor (VEGF), epidermal growth factor (EGF), transforming growth factor-β (TGF-β), and insulin-like growth factor-1 (IGF-1) released after platelet activation [8, 9].

The application of cell-based therapy as an alternative to treat medical disorders has skyrocketed in recent years. Specifically, stem cell therapy has been used to manage various dermatological disorders [10, 11], one of which is alopecia. Stem cells (SCs) are reported to play a significant role in initiating the hair growth cycle and hair remodeling [11]. Accumulating evidence suggests that adipose tissue-derived stem cells (ADSC) may be responsible for the secretion of cytokines, chemokines, and specific growth factors, namely bFGF, VEGF, IGF-1, hepatocyte growth factor (HGF), and TGF-β1 [12, 13]. These molecules trigger the process of cell repair through angiogenesis, immunomodulation, cell differentiation, and proliferation. In the scalp, these components exert a paracrine effect on surrounding cells and tissues, which stimulate hair follicle growth, modulate hair growth dynamics, regulate hair follicle size through angiogenesis, and help retain the anagen phase [11]. This finding is also supported by recent studies that observed the potential efficacy of ADSCs and their derivatives as an alternative management for alopecia. The use of adipose-derived stem cells conditioned media (ADSC-CM) and constituent extract (ADSC-CE) demonstrated substantiate results in terms of hair count and hair diameter [14–18].

Nevertheless, there have been limited randomized, placebo-controlled trials in the Asian population that aim to observe the efficacy of ADSC-CM therapy in AGA. Therefore, we conducted a randomized clinical trial to evaluate the effectiveness and tolerability of ADSC-CM for treating male AGA in combination with minoxidil. Based on existing evidence, we believe that this combination could serve as a prospective and safe agent to induce hair growth, particularly in male patients with AGA.

## Materials and methods

### Design overview

This research was a double-blind, randomized clinical trial in AGA patients that aimed to compare the improvement in the quality of clinical parameters between scalp sections treated with ADSC + minoxidil and scalp sections treated with 0.9% NaCl + minoxidil. In total, 62 male adults were screened, and 37 participants were eventually chosen to participate. All subjects were identified using numbers determined at the time of recruitment. Individuals who met the inclusion criteria were randomized into two treatment groups: concentrated ADSC-CM or non-concentrated ADSC-CM. Randomization was conducted by an unaffiliated party using *random.org*. The investigators in charge of enrolling the participants and conducting the assessments were completely blinded to the randomization procedure throughout the entire study. Additionally, those who administered the injection were kept in the dark regarding the outcomes of the tests as well as treatment groups.

### Study setting and participants

Our study was conducted in the dermato-venereology outpatient clinic of Dr. Cipto Mangunkusumo Hospital for 3 months, from October 2021 to December 2021. The authors received ethical approval from the Health Research Ethics Committee—Faculty of Medicine, Universitas Indonesia/Dr. Cipto Mangunkusumo Hospital with decision number KET-219/UN2.Fl/ETIK/PPM.00′00/2020 prior to the study. Male AGA patients between the ages of 25 and 59 years old whose clinical presentation met Hamilton-Norwood criteria grades II to VI were included in the study. Cessation of anti-androgens and minoxidil treatment for at least 1 month was mandatory. The following were the criteria for exclusion:

1. patients with hair loss other than AGA
2. patients receiving 5-alpha reductase inhibitor therapy;
3. patients who had received growth factor treatment (platelet-rich plasma or microneedling) for at least 6 months prior to the study; and
4. history of hypertrophic scars or blood clotting disorders.

Study participants were split into two treatment groups: 20 received concentrated ADSC-CM, and 17 received non-concentrated ADSC-CM on the treated scalp section. The sample size was calculated using Federer’s formula. Patients were dropped out of the study if they experienced severe side effects, withdrew, or failed to follow the appointed protocol.

### Production of ADSC-CM

ADSC-CM was produced by the Stem Cell Medical Technology Unit of Dr. Cipto Mangunkusumo Hospital. ADSC-CM used in this study was allogeneic, formed by the culture of human mesenchymal stem cells (MSC). These mesenchymal stem cells originated from adipose tissue which has been screened previously for infectious pathogens. After successful isolation of MSCs, these stem cells were multiplied in a normoxic condition until it reached the fifth passage, with a culture confluence of at least 80%. The conditioned media were then obtained by removing the stem cells.

Following a round of centrifugation to remove existing debris, ADSC-CM was stored frozen at -20 °C and slowly thawed at 4 °C overnight before undergoing filtration using a 0.22 µm syringe filter. Further processing for concentrated ADSC-CM was conducted using the Spin-X UF 500 Concentrator (Corning). Both non-concentrated and concentrated ADSC-CM was subjected to sterility tests to exclude the presence of bacteria, fungi, and mycoplasma endotoxins, as well as a potency test to evaluate the number of proteins and growth factors.

### Intervention method

**Fig. 1:**
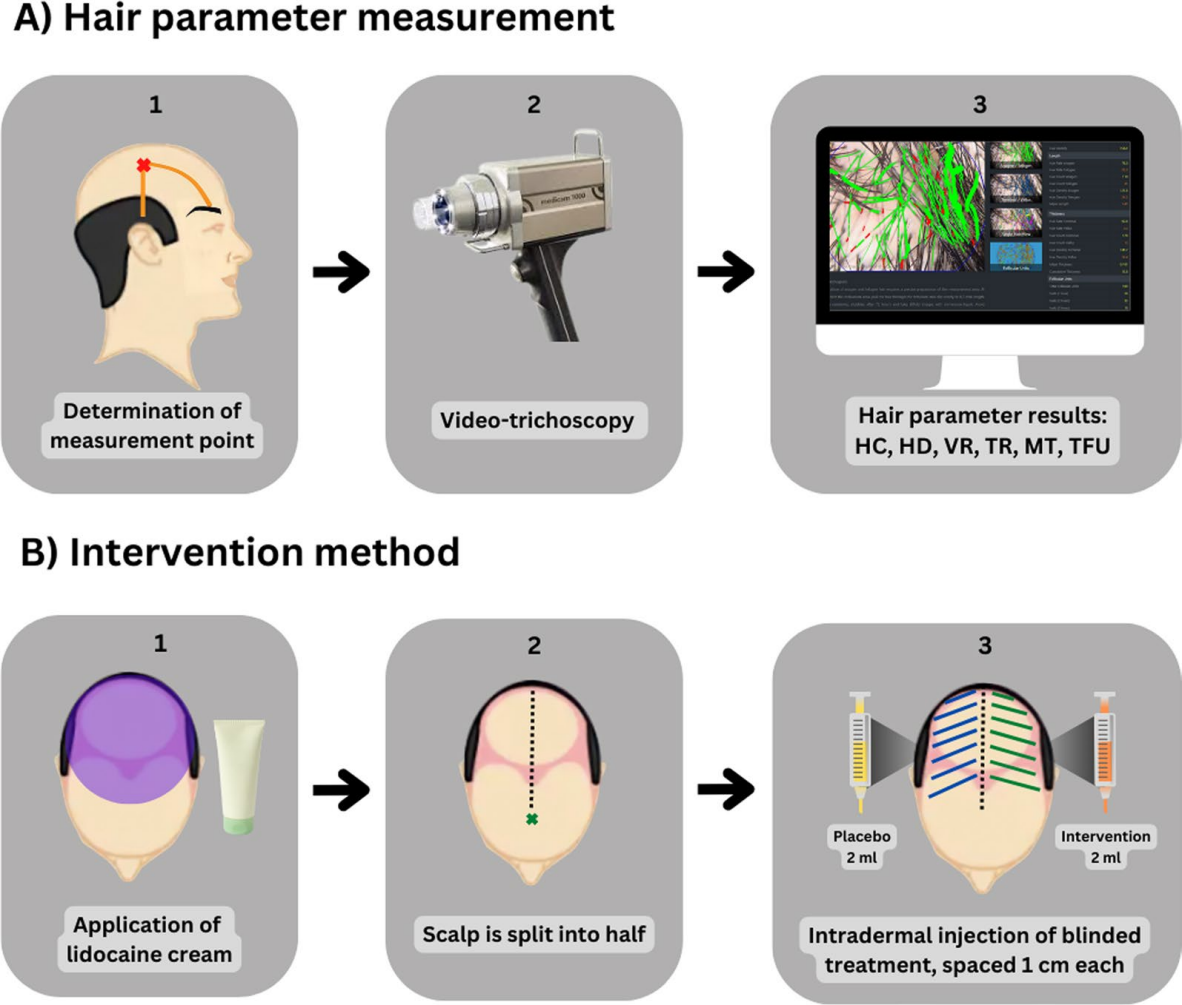
**A** Hair parameters were assessed using Fotofinder^®^ medicam 1000 s video-trichoscopy (2) at the meeting point (1) of the highest part of the ears and the medial aspect of each eyebrow (indicated by red “x” marks) on both sides of the scalp. The findings were available for immediate viewing on a computer linked to the device (3). **B** After the administration of lidocaine cream, the scalp was divided into two parts. The placebo was applied to one side of the scalp and the treatment solution was to the other. Intradermal injections were given at 1 cm distances from the temple to the crown (hatched in blue and green lines)

Summary of intervention method is illustrated in Fig. 1. Study participants must wash their hair before the intervention. The test area was cleaned using 70% alcohol, given 4% lidocaine cream, and was left for 30–60 min before the procedure began. Two concentrations of ADSC-CM were given to the subjects, concentrated and non-concentrated. Subjects’ scalps were divided into two sections, with one receiving 2 ml of concentrated or non-concentrated ADSC-CM and the other receiving 2 ml of NaCl 0.9% as control. These agents were administered intradermally from the frontal part of the head toward the vertex using a 30G needle, with each injection spaced approximately 1 cm apart. Afterward, the injection area is massaged to increase the penetration of the liquid through the epidermis. Over 6 weeks, this regimen was administered intradermally every 2 weeks. Subjects were instructed to apply 1 ml of topical 5% minoxidil twice daily until the final treatment. Self-documentation of minoxidil use was written in a daily log book reviewed by the researchers during each session.

### Outcomes and measurements

Subjective evaluation is the patient satisfaction scale using a linear analog scale with a scale of 1–7 (1 = no result, 7 = very satisfactory outcome) and was evaluated at the last treatment session. Objective clinical assessment was conducted every 2 weeks until the sixth week. We used Fotofinder^®^ medicam 1000 s video-trichoscopy (FotoFinder Systems GmbH, Germany) to monitor the following parameters: hair count (HC), hair density (HD), vellus rate (VR), terminal rate (TR), mean thickness (MT) and total follicular units (TFU). A point of intersection between the medial part of the eyebrows and the uppermost side of the ears, on which a substantial degree of baldness was observed, was determined as the place of measurement. Macroscopic hair growth was documented using iPhone XS^®^ (Apple Inc., Cupertino, CA, USA) and Fotofinder^®^ medicam 1000 s video-trichoscopy. Only one evaluator consistently performed these assessments.

### Statistical analysis

The data were processed with SPSS^®^ Statistics v.24 (IBM^®^, NY, USA). Continuous data (anthropometric measures, age, onset of alopecia, trichoscan results) are presented as mean ± SD, if normally distributed, or median (min–max) if otherwise. On the other hand, categorical data (Hamilton–Norwood grade, patients' previous medical history and treatments, smoking status, current complaints, patient satisfaction) are described as frequency (percentage).

Shapiro–Wilk’s test was used to test the normality assumption. Intergroup comparisons of baseline characteristics were performed using the independent-samples *T*-test or Mann–Whitney *U* test for nonparametric data, and the chi-square test or Fisher’s exact test for nonparametric data. Changes over time were analyzed using paired samples *T*-test or Wilcoxon’s signed-rank test. Percentage of change in hair parameters were compared between groups using one-samples ANOVA or Kruskal–Wallis test. The result was statistically significant if the *p* value was < 0.05.

## Results

### Baseline characteristics

**Fig. 2:**
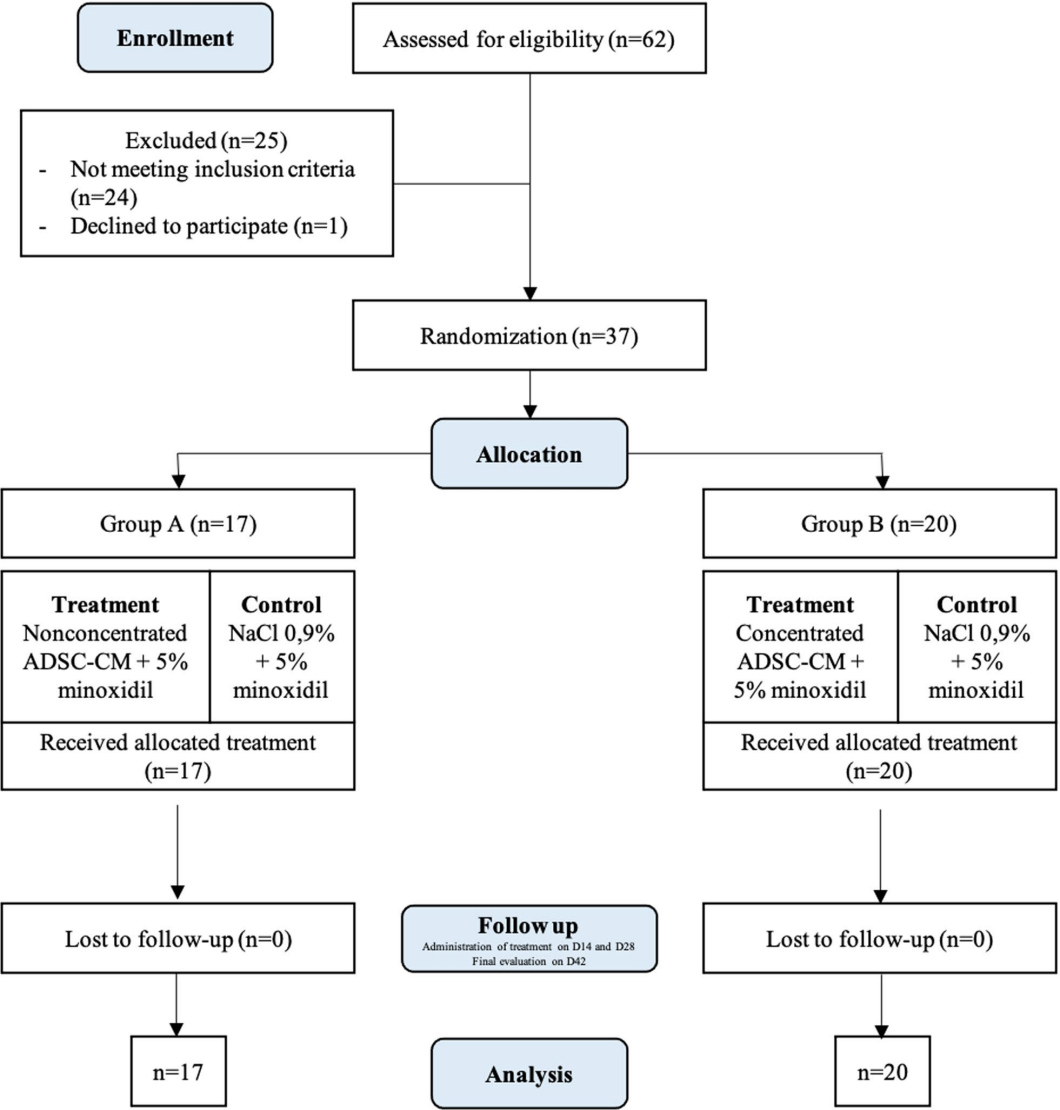
CONSORT flow diagram

A total of 62 male adults were screened, and 37 participants were accepted to the study according to the inclusion and exclusion criteria (Fig. 2). Seventeen participants (45.95%) were put into the non-concentrated group upon randomization, while 20 subjects (54.05%) were assigned to the concentrated ADSC-CM group. None of the study participants withdrew from the study or were lost to follow-up; therefore, 37 subjects completed the trial as planned. A complete comparison of the groups’ sociodemographic characteristics at baseline is further elaborated in Table 1. All enrolled individuals were males with an average age of 36.78 years. AGA manifested in patients approximately 6.95 years before the intervention.

**Table 1:** Subject characteristics based on treatment groups

Characteristics	Non-concentrated ADSC-CM (*n* = 17)	Concentrated ADSC-CM (*n* = 20)
Age (years)	36.05 ± 6.02	37.40 ± 10.35
Weight (kg)	77.52 ± 14.86	78.5 ± 12.55
Height (cm)	170.71 ± 6.41	171.85 ± 6.36
Waist circumference (cm)	93.82 ± 13.53	97.70 ± 15.30
BMI (kg/m^2^)	26.57 ± 4.04	26.45 ± 4.01
Hamilton–Norwood grade		
III	2 (11.8)	1 (5.0)
III vertex	1 (5.9)	3 (15.0)
IV	2 (11.8)	2 (10.0
IVa	3 (17.6)	5 (25.0)
V	2 (11.8)	2 (10.0)
Va	2 (11.8)	3 (15.0)
VI	5 (29.4)	4 (20.0)
Onset of alopecia (years)	7.67 ± 5.00	6.35 ± 3.11
Family history of alopecia		
No history	2 (11.8)	3 (15.0)
Maternal	7 (41.1)	7 (35.0)
Paternal	3 (8.1)	8 (40.0)
Others	10 (27.0)	8 (40.0)
Multiple family members	5 (13.5)	8 (40.0)
Medical history		
Hypertension	4 (23.5)	3 (15.0)
Dyslipidemia	4 (23.5)	6 (30.0)
COVID-19	3 (17.6)	5 (25.0)
Smoking status	5 (29.4)	4 (20.0)
Current complaints		
No symptoms	2 (5.4)	1 (5.0)
Cosmetic disturbance	14 (82.3)	13 (65.0)
Low self-esteem	1 (5.9)	4 (20.0)
Itch	11 (64.7)	10 (50.0)
Others	16 (94.1)	10 (50.0)
Previous therapy for alopecia		
No therapy	4 (23.5)	6 (30.0)
Topical minoxidil	6 (35.2)	6 (30.0)
Non-pharmaceutical agents	9 (52.9)	8 (40.0)
Oral finasteride	3 (17.6)	1 (5.0)
Surgical intervention	2 (11.7)	1 (5.0)
Others	3 (17.6)	3 (15.0)
Current medication history		
No medication	9 (52.9)	9 (45.0)
Systemic medication	5 (29.4)	7 (35.0)
Vitamins, supplements	2 (5.4)	4 (20.0)
Others	1 (5.9)	2 (10.0)

Participants of the concentrated group had a higher mean of age, weight, height, and waist circumference, although no significant intergroup difference was observed. On the other hand, we discovered a higher average of onset of disease in the non-concentrated group (*p* = 0.570). All patients came to our clinic with multiple complaints; however, twenty-seven patients (72.97%) experienced a sense of cosmetic disturbance, making it the most frequent complaint. Five patients (13.51%) denied any family history of alopecia. The previous treatment for alopecia most employed by study participants was non-pharmaceutical topical agents (45.94%), followed by topical minoxidil (37.83%). Almost one-half of the participants were not currently on any routine medication (48.64%). Based on these results, we concluded that there were no significant differences between the treatment groups regarding sociodemographic and clinical characteristics.

All hair parameters (HC, HD, VR, TR, MT, and TFU) were subjected to baseline analysis using one-way ANOVA. Aside from TFU, no statistically significant differences (*p* > 0.05) between the groups were observed. These results can be observed in Table 2.

**Table 2:** Baseline analysis on hair parameters between groups

Parameters	Non-concentrated ADSC-CM (*n* = 17)		*p*^a^	Concentrated ADSC-CM (*n* = 20)		*p*^a^
	Intervention	Control		Intervention	Control	
HC	124.52 ± 61.97	164.76 ± 57.76	0.059	130.50 ± 45.86	125.65 ± 39.19	0.721
HD	137.99 ± 68.47	182.73 ± 64.35	0.058	144.47 ± 50.78	138.61 ± 42.74	0.695
VR	43.05 ± 19.84	36.71 ± 20.92	0.390	38.66 ± 19.20	36.71 ± 20.92	0.401
TR	57.23 ± 19.48	63.28 ± 20.92	0.371	61.33 ± 19.20	56.61 ± 18.22	0.430
MT	0.044 ± 0.08	0.046 ± 0.00	0.560	0.046 ± 0.00	0.045 ± 0.01	0.627
TFU	90.29 ± 35.32	115.29 ± 28.56	0.030	94.95 ± 21.74	93.00 ± 23.10	0.785

*HC* Hair count, *HD* Hair density, *VR* Vellus rate, *TR* Terminal rate, *MT* Mean thickness, *TFU* Total follicular units

^a^*p* values according to one-way ANOVA

### Hair growth parameters

#### Non-concentrated ADSC-CM

Overall, HC, HD, TR, VR, and MT improved over 6 weeks, both in the treatment and placebo side (Table 3). Changes over time, from baseline to week sixth, were statistically significant in both groups (*p* < 0.05). In contrast, we witnessed a dramatic decrease of TFU in the placebo side (*p* = 0.005). The same phenomenon was also observed in the treatment side but it was statistically insignificant (*p* = 0.847). Furthermore, half-side comparison revealed that the treatment side had more superior changes from baseline, although none of the variables reached statistical significance.

**Table 3:** Changes from baseline to week 6 using trichoscan examination

	Observed value						Changes from baseline		
	Placebo			Intervention			Placebo	Intervention	*p*^b^
	Week 0	Week 6	*p*^a^	Week 0	Week 6	*p*^a^			
Non-concentrated ADSC-CM (*n* = 17)									
HC	164.76 ± 57.76	321.47 ± 120.09	**0.006**	132.29 ± 68.86	317.17 ± 118.89	**0.000**	156.70 ± 59.79	184.88 ± 119.63	0.563
HD	182.73 ± 64.35	352.71 ± 117.14	**0.001**	137.99 ± 68.47	351.11 ± 131.62	**0.000**	169.97 ± 158.95	213.12 ± 146.57	0.417
VR	36.71 ± 20.92	22.04 ± 12.98	**0.003**	43.05 ± 19.84	18.54 ± 11.76	**0.001**	− 14.67 ± 15.81	− 24.51 ± 22.53	0.150
TR	63.28 ± 20.92	77.95 ± 12.98	**0.003**	57.23 ± 19.48	81.42 ± 11.75	**0.001**	14.67 ± 15.81	24.19 ± 22.21	0.160
MT	0.041 ± 0.00	0.056 ± 0.01	**0.000**	0.042 ± 0.08	0.062 ± 0.01	**0.004**	0.016 ± 0.01	0.024 ± 0.01	0.120
TFU	115.29 ± 28.56	90.29 ± 35.32	**0.005**	178.23 ± 37.38	176.29 ± 41.74	0.847	− 25.00 ± 31.21	− 1.94 ± 40.86	0.220
Concentrated ADSC-CM (*n* = 20)									
HC	125.65 ± 39.19	310.10 ± 104.70	**0.000**	130.50 ± 45.86	310.15 ± 104.70	**0.000**	184.45 ± 103.92	174.65 ± 119.89	0.784
HD	138.61 ± 42.74	343.30 ± 115.91	**0.000**	144.47 ± 50.78	337.82 ± 16.58	**0.000**	204.69 ± 14.45	193.34 ± 132.73	0.774
VR	43.69 ± 18.23	16.52 ± 7.66	**0.000**	38.66 ± 19.20	15.35 ± 7.75	**0.000**	− 27.16 ± 18.94	− 23.31 ± 20.16	0.568
TR	56.61 ± 18.22	83.47 ± 7.66	**0.000**	61.33 ± 19.20	84.65 ± 7.75	**0.000**	26.86 ± 18.81	23.32 ± 20.15	0.569
MT	0.045 ± 0.01	0.069 ± 0.13	**0.000**	0.046 ± 0.01	0.074 ± 0.01	**0.000**	0.024 ± 0.01	0.027 ± 0.01	0.577
TFU	93.00 ± 23.10	171.85 ± 35.79	**0.000**	94.95 ± 21.74	174.60 ± 38.57	**0.000**	78.85 ± 41.63	79.65 ± 46.34	0.955

Bold values indicate statistical significance

*HC* Hair count, *HD* Hair density, *VR* Vellus rate, *TR* Terminal rate, *MT* Mean thickness, *TFU* Total follicular units

^a^*p* values according to paired samples *T*-Test or Wilcoxon’s signed-rank test

^b^*p* values according to independent-samples *T*-test

#### Concentrated ADSC-CM

Comparisons between placebo and treatment sides are demonstrated in the lower portion of Table 3. Similarly, both the placebo and intervention sides recorded a positive and statistically significant changes after 6 weeks of treatment in all hair parameters (*p* < 0.05). Contrary to the non-concentrated ADSC-CM group, after 6 weeks, TFU increased in the placebo (*p* = 0.005) and intervention (*p* = 0.000) sides of the scalp. Administration of placebo, however, was able to induce a greater change from baseline compared to intervention in almost all parameters except TFU and MT. Nonetheless, none of these changes could be deemed statistically significant.

#### Comparison between treatment groups

**Fig. 3:**
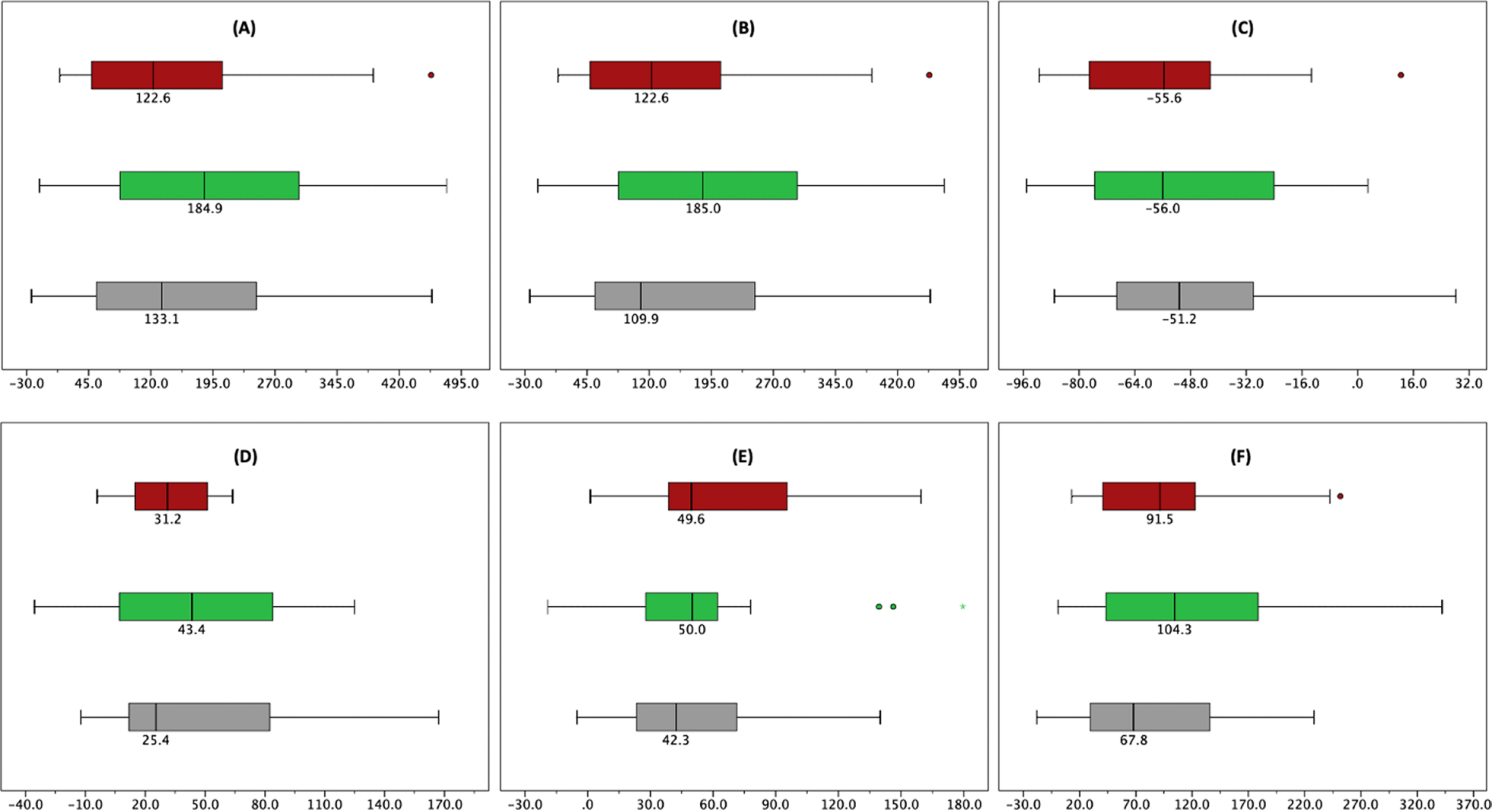
Percentage change between treatment groups compared to baseline, presented as median (min–max). Brown filled square denotes concentrated ADSC-CM (*n* = 20). Green filled square denotes non-concentrated ADSC-CM (*n* = 17). Gray filled square denotes placebo (*n* = 37). **A** HC (*p* = 0.562)^1^, **B** HD (*p* = 0.534)^1^, **C** VR (*p* = 0.774)^1^, **D** TR (*p* = 0.742), **E** MT (*p* = 0.540)^1^, **F** TFU (*p* = 0.451)^1^. ^1^*p* values according to one-samples ANOVA or Kruskal–Wallis test

Figure 3 describes the percentage change in hair parameters compared to baseline between the treatment groups. Comparisons between groups revealed that the non-concentrated ADSC-CM group had the highest percentage of increase in HC (184.9), HD (185.0), TR (43.4), MT (50.0), and TFU (104.3), as well as the highest decline in VR (− 56.0). Nonetheless, these results were statistically insignificant.

### Patient satisfaction

In the last treatment session, patients filled in a 7-point scale regarding the improvement of their hair post-intervention. Thirty patients (81.08%) claimed that they were very satisfied (7 points) with the outcome, while 7 others (18.91%) were satisfied (6 points). No significant intergroup differences were found.

### Safety evaluation

Most patients complained of mild local irritation, itchiness, and folliculitis during minoxidil treatment. One participant developed contact dermatitis after 2 weeks of minoxidil usage. This patient experienced remission of adverse effects after treatment with topical steroids and oral antihistamines. One patient presented with excessive facial hair growth but did not receive any medication. Pain, itchiness, and redness around the treatment area were the most reported post-injection side effects. These effects were still tolerable and resolved soon after.

## Discussion

In this study, we aimed to identify whether intradermal administration of ADSC-CM could stimulate hair regeneration in AGA. All 37 patients that enrolled in this study were men (100%), as AGA is more prevalent in males [1, 3, 19, 20]. Participants’ average age was 36.78 years, similar to a previous study in India [19] and China [20]. It is widely known that the AGA usually manifests between 30 and 40 years [1–3]. This study found that the average onset of alopecia is 30 years old, with an overall range of 18–49 years of age. Dyslipidemia was the most frequent coexisting systemic disease in this study, supported by studies that found that dyslipidemia is indeed more common in AGA compared to the normal population [21–23]. AGA could negatively affect a person’s appearance and overall mental well-being [2, 5] as almost three-quarters of the patients in this study experienced a sense of cosmetic disturbance.

Treatment of AGA is quite challenging. Available treatment modalities, while abundant, fail to provide a lasting effect on hair growth [4, 6, 24–29]. Only long-term treatment using a combination of several modalities could generate consistent hair growth, but this positive impact will gradually deteriorate if not appropriately maintained [29]. Furthermore, treatment outcomes also heavily depend on age, severity, and compliance [26]. Hence, there is an enormous demand for effective solutions for hair loss.

Autologous platelet-rich plasma (A-PRP) and adult SCs such as adipose-derived mesenchymal stem cells (AD-MSCs) and human follicle stem cells (HFSCs) are proposed as minimally invasive alternative treatments for AGA. The antiapoptotic effects of A-PRP have been suggested as one of the primary elements encouraging hair growth by extending the survival of dermal papilla cells (DPCs) throughout the hair cycle, stimulating HFSC differentiation, and lengthening the anagen phase. Additionally, it enhanced the perifollicular vascular plexus by raising levels of angiogenic factors [30–32].

Recent studies have shown that A-PRP as an AGA treatment is clinically beneficial. In a recent study by Gentile et al. the improvement in hair density for A-PRP was 28 ± 2% hairs/cm^2^ 23 weeks after the third infiltration compared with placebo. In the same study, the improvement in hair density for HFSC treatment was 29 ± 5% hairs/cm^2^ 23 weeks after the second infiltration and less than a 1% increase in hair density for the placebo area [31, 32]. Gkini et al. demonstrated the efficacy of PRP injection, with three sessions separated by 3 weeks, in 22 individuals with AGA in a prospective cohort. The patients’ hair density showed a significant improvement at 6 weeks and up to 1 year after initial treatment [30].

Few clinical trials have investigated the effects of ADSC-based therapies on the hair cycle in humans. An observational study in 27 females with AGA who were treated with single ADSC-CM intradermal injection resulted in elevated hair density and thickness [15]. Prior study conducted by Fukuoka et al. in 22 males and females with alopecia illustrated an increase in hair density and hair count effect after 6 injections of ADSC-CM [14, 16]. Similar to our trial, this study incorporated the first line therapy of AGA, in this case oral finasteride, with ADSC-CM, although only in male participants. Although the duration of these studies was generally longer than our trial, which allowed the investigators to observe full hair growth potential, these studies did not include a control group for comparison. As far as we know, only one study included a control group, which enrolled 29 men, applied ADSC-CE for 16 weeks and discovered favorable outcomes [18].

**Fig. 4:**
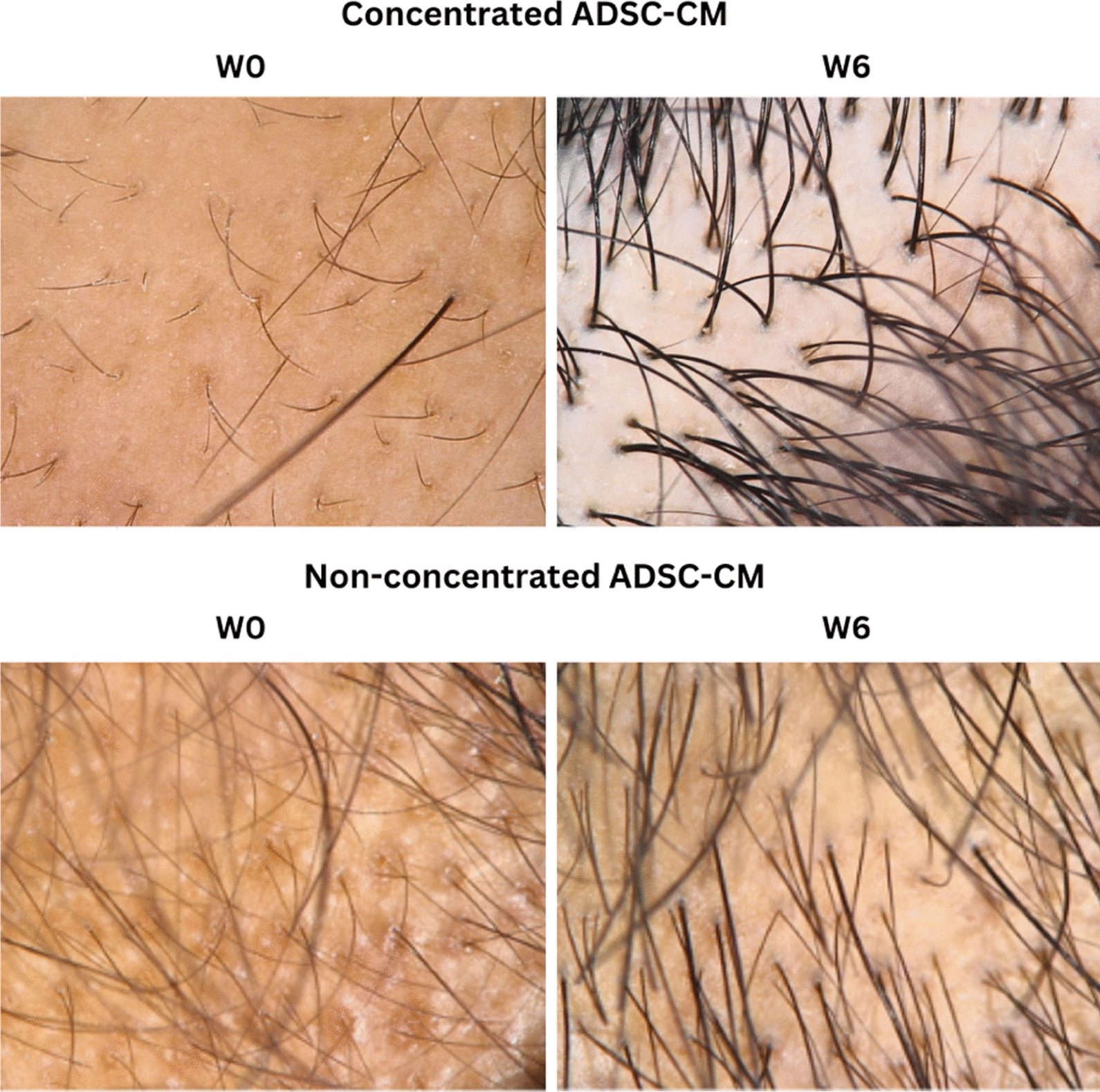
Microscopic changes observed using trichoscopy at baseline and 6 weeks after ADSC-CM and minoxidil application

**Fig. 5:**
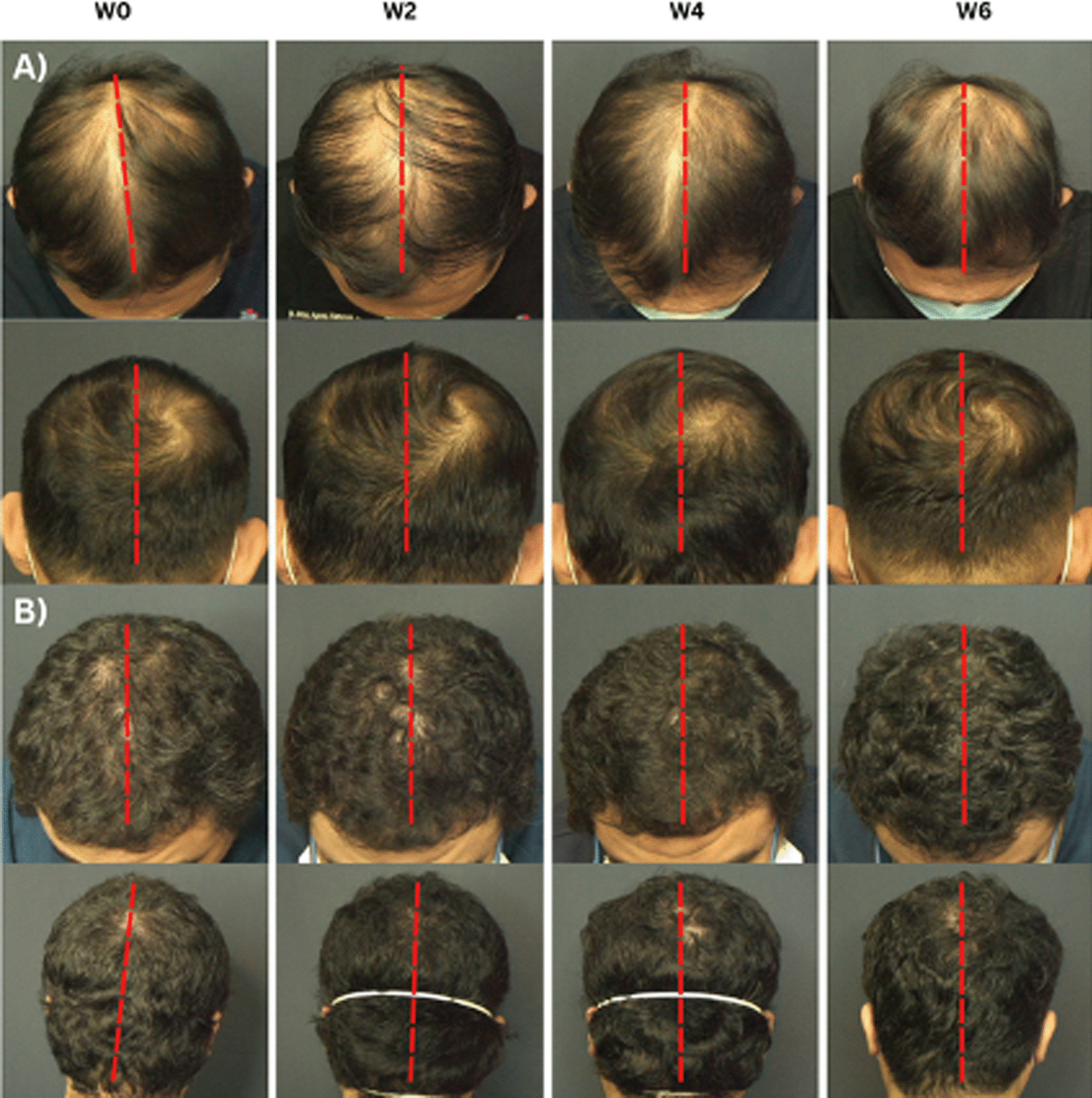
Macroscopic changes were taken using Fotofinder^®^ medicam 1000 s video-trichoscopy at baseline, 2 weeks, 4 weeks, and 6 weeks after ADSC-CM and minoxidil application in the intervention side (left) and the placebo side (right), which are separated by dotted lines. Pictures **A** were documented from a patient belonging to the concentrated group, while **B** was from the non-concentrated group

We believe that objective evaluation of hair growth is vital to determine the efficacy of our treatment regimen. Hence, we utilized a non-invasive video-trichoscopy device that is able to measure various hair parameters in real time. To our knowledge, we are the only study that included parameters such as VR, TR, MT, and TFU in our data. We observed a significant improvement not only microscopically, as shown in Fig. 4, but also in the macroscopic appearance of hair, particularly in the vertex area (Fig. 5). In addition, ADSC-CM was generally well-tolerated by all participants as no severe side effects were experienced.

Our results demonstrated that concentrated ADSC-CM successfully promoted the increase of HC, HD, TR, MT, and TFU as well as decreased the number of VR compared to baseline. Although patients in the non-concentrated ADSC-CM group also generated similar positive outcomes in hair parameters, we saw a decline in TFU on both the placebo and treatment sides of the scalp. We suspect that this is due to the use of topical minoxidil, which is known for causing initial hair loss after treatment is initiated. The injection of non-concentrated ADSC-CM on the treatment side may have minimized the hair loss, which would account for the greater reduction on the placebo side.

Much to our surprise, we found that the majority of hair growth parameters also improved on the placebo side, and there was no statistically significant difference between the groups. Analysis using another statistical method, mixed model linear regression, also showed similar insignificant difference between the groups (Additional file 1: Table S4). The same phenomenon was observed by Fukuoka et al. whose research method was a half-comparison study [14]. There are a number of speculated pathways that might lead to this specific outcome. First, it is believed that microlesions due to intradermal injections stimulate hair growth. These microlesions promote vasodilation, induce wound healing through collagen and keratin production, and trigger platelets and neutrophils to produce various growth factors such as PDGF, VEGF, transforming growth factor (TGF), and fibroblast growth factor (FGF) which induce repair of surrounding skin structures [24, 25, 34]. However, as these injuries are considered too small to exert a meaningful effect, another theory suggested that alteration of the electrical potential due to the interaction between cell membranes and needles could perhaps be the reason [25].

Second, we hypothesize that ADSC-CM might have diffused to the placebo-treated side of the scalp. This could explain why in both sections of the concentrated ADSC-CM group, all parameters increased simultaneously, with very identical results. It is possible that larger concentrations of ADSC-CM enabled a more optimal spread of growth factors, causing the placebo-treated side of concentrated ADSC-CM to have a higher mean in all hair parameters than the non-concentrated ADSC-CM group. Dissemination of growth factors in ADSC-CM through local circulation exert a paracrine effect on the surrounding tissues and cells [33]. These growth factors favorably control hair follicle activity and promote hair development [33, 35]. During the anagen phase, expression of growth factors VEGF promotes hair growth and follicular expansion by triggering angiogenesis [33, 36, 37].

To date, our study is one of the first clinical trials to evaluate the efficacy of ADSC-CM combined with minoxidil in AGA. The rationale behind our decision to incorporate 5% topical minoxidil with ADSC-CM in our treatment regimen stems from the fact that 5% topical minoxidil is widely recognized as the first-line standard therapy for AGA. As the safety and efficacy of ADSC-CM use for AGA is currently limited, given the preliminary nature of our pilot study conducted in Indonesia, we found it essential to retain minoxidil as the standard therapy. Still, we believe that ADSC-CM possesses the potential as adjuvant therapy. It is also the first to apply two different concentrations of ADSC-CM, as we believed the culture medium used during production might have diluted the growth factors. Yet, we could not find any statistically significant difference between the use of concentrated and non-concentrated ADSC-CM. Disruption in the expression of growth factors might be a cause, which affects the hair cycle differently depending on age and sex [33, 36].

We believe that outcomes of ADSC-based treatments for AGA vary depending on formulation type, presence of other treatments, and mode of administration. Pre-existing studies which applied different formulations, treatment duration, and study methods have generated variable results [14–18]. As a result, in clinical practice, personalizing the best concentration, dosage, and frequency of ADSC-based therapy is crucial to achieve the best treatment response with fewest possible adverse effects [16]. This phenomenon also calls for more standardized trials with larger samples.

There are certain limitations to our research. First, since the study duration was 6 weeks long, maximum hair growth could not be adequately measured and documented. Even though the ADSC-CM used in our study did not cause any substantial side effects, the short duration of the study may not be able to assure its long-term safety. Furthermore, our results may not be generalizable as our sample size was relatively small. Lastly, the half-scalp comparison study design and the use of minoxidil as standard therapy might have obscured the therapeutic potential of ADSC-CM.

Despite these shortcomings, our research still possesses several strengths. This is the first randomized, double-blind clinical trial in Indonesia to assess the potential of ADSC-CM combined with minoxidil for hair regrowth in AGA. Hair regrowth was also measured objectively using trichoscan, which produced more reliable quantitative assessments compared to a photograph-based analysis by an investigator or self-assessments by study participants, both of which have been employed in earlier studies. All patients’ photographic views were taken on the same fixed area of the scalp, including the same light and distance from the lens. All evaluations each week are performed by the same person. As this research was conducted during the COVID-19 pandemic, researchers strongly implemented strict safety and health measures.

## Conclusion

The use of ADSC-CM combined with minoxidil for 6 weeks significantly improved almost all hair parameters in all treatment groups. There were no severe topical or systemic side effects related to the use of ADSC-CM for hair growth in humans. We believe that direct application of ADSC-CM to the scalp could be a potential agent. However, to confirm the beneficial effects of ADSC-CM on hair growth and explain the mechanisms responsible for the action of ADSC-CM in humans, similar research with extensive populations, longer duration, and different study designs may be required.

### Supplementary Information

**Additional file 1: Table S4.** Estimates of fixed effects using generalized linear mixed model regression.

## Data Availability

All data generated or analyzed during this study are included in this article. Further enquiries can be directed to the corresponding author.

## Abbreviations

AGA: Androgenetic alopecia
HRQOL: Health-related quality of life
HTE: Hair tissue engineering
PRP: Platelet-rich plasma
HF-MSCs: Human follicular mesenchymal stem cells
HTR: Hair tissue regeneration
bFGF: Basic fibroblast growth factor
PDGF: Platelet-derived growth factor
VEGF: Vascular endothelial growth factor
EGF: Epidermal growth factor
TGF-β: Transforming growth factor-β
IGF-1: Insulin-like growth factor-1
SC: Stem cell
HGF: Hepatocyte growth factor
ADSC: Adipose tissue-derived stem cells
ADSC-CM: Adipose tissue-derived stem cells conditioned media
ADSC-CM: Adipose tissue-derived stem cells constituent extract
MSC: Mesenchymal stem cells
HC: Hair count
HD: Hair density
VR: Vellus rate
TR: Terminal rate
MT: Mean thickness
TFU: Total follicular units
A-PRP: Autologous platelet-rich plasma
AD-MSC: Adipose-derived mesenchymal stem cells
HFSC: Human follicle stem cell
DPC: Dermal papilla cell
TGF: Transforming growth factor
FGF: Fibroblast growth factor
