# Supplementary material for: Combination of adipose-derived stem cell conditioned media and minoxidil for hair regrowth in male androgenetic alopecia: a randomized, double-blind clinical trial
Source: Stem Cell Res Ther. 2023 Aug 21;14:210. doi: 10.1186/s13287-023-03440-2 (PMC10441691; doi:10.1186/s13287-023-03440-2)
Supplement: Supplementary file 1 — Additional file 1: Table S4. Estimates of fixed effects using generalized linear mixed model regression. [file 13287_2023_3440_MOESM1_ESM.docx]

Table 4. Estimates of fixed effects using generalized linear mixed model regression

| **Non-concentrated ADSC-CM (n=17)** | | | |
| --- | --- | --- | --- |
| **Week^a^** | | **95% CI (Lower⎯upper bound)** | ***P*^1^** |
|  | HC | [(-255.64)⎯(-129.64)] | 0.000 |
|  | HD | [(-281.96)⎯(-144.28)] | 0.000 |
|  | VR | [12.90⎯36.12] | 0.000 |
|  | TR | [(-6.79)⎯25.83] | 0.000 |
|  | MT | [(-0.03)⎯(-0.01)] | 0.000 |
|  | TFU | [(-110.72)⎯(-61.26)] | 0.000 |
| **Type of intervention^b^** | | **95% CI [Lower⎯upper bound]** | ***P*^1^** |
|  | HC | [(-67.92)⎯92.02] | 0.761 |
|  | HD | [(-85.45)⎯88.64] | 0.970 |
|  | VR | [(-5.15)⎯12.17] | 0.416 |
|  | TR | [(-12.13)⎯5.17] | 0.419 |
|  | MT | [(-0.16)⎯0.00] | 0.207 |
|  | TFU | [(-25.74)⎯29.62] | 0.887 |
| **Concentrated ADSC-CM (n=20)** | | | |
| **Week^c^** | | **95% CI [Lower⎯upper bound]** | ***P*^1^** |
|  | HC | [(-62.27)⎯72.17] | 0.000 |
|  | HD | [(-249.65)⎯(-137.03)] | 0.000 |
|  | VR | [14.63⎯32.79] | 0.000 |
|  | TR | [(-32.41)⎯(-14.22)] | 0.000 |
|  | MT | [(-0.03)⎯(-0.01)] | 0.000 |
|  | TFU | [(-99.07)⎯(-60.22)] | 0.000 |
| **Type of intervention^d^** | | **95% CI [Lower⎯upper bound]** | ***P*^1^** |
|  | HC | [(-225.65)⎯(-123.84)] | 0.882 |
|  | HD | [(-68.93)⎯79.90] | 0.882 |
|  | VR | [(-3.75)⎯6.10] | 0.633 |
|  | TR | [(-6.11)⎯3.75] | 0.631 |
|  | MT | [(-0.01) ⎯ 0.00] | 0.251 |
|  | TFU | [26.57⎯21.07] | 0.816 |
| HC: hair count, HD: hair density, VR: vellus rate, TR: terminal rate, MT: mean thickness, TFU: total follicular units  **^1^**Analysis using generalized mixed model regression, significant if P<0.05 | | | |
| **^a^**Difference between week 0 and 6 in the non-concentrated group | | |  |
| **^b^**Difference between placebo and intervention in the non-concentrated group | | | |
| **^c^**Difference between week 0 and 6 in the concentrated group | | |  |
| **^d^**Difference between placebo and intervention in the concentrated group | | | |
